# Theranostic Contribution of Extracellular Matrix Metalloprotease Inducer-Paramagnetic Nanoparticles Against Acute Myocardial Infarction in a Pig Model of Coronary Ischemia-Reperfusion

**DOI:** 10.1161/CIRCIMAGING.121.013379

**Published:** 2022-06-09

**Authors:** Rafael Ramirez-Carracedo, Marcelo Sanmartin, Amadeo Ten, Ignacio Hernandez, Laura Tesoro, Javier Diez-Mata, Laura Botana, Karina Ovejero-Paredes, Marco Filice, Angel Alberich-Bayarri, Luis Martí-Bonmatí, Carlota Largo-Aramburu, Marta Saura, Jose Luis Zamorano, Carlos Zaragoza

**Affiliations:** 1 Unidad Mixta de Investigación Cardiovascular, Departamento de Cardiología, Universidad Francisco de Vitoria, Hospital Ramón y Cajal (IRYCIS), Madrid, Spain (R.R.-C., I.H., L.T., J.D.-M., L.B., C.Z.).; 2 Departamento de Cardiología, Hospital Universitario Ramón y Cajal (IRYCIS), Madrid, Spain (M. Sanmartin, J.L.Z.).; 3 Instituto de Investigación de salud La Fe, Grupo de Investigación Biomédica (GIBI230-PREBI). Nodo de Imagen La Fe en la Red de Imagen Biomédica (ReDIB) de Infraestructuras Científicas Técnicas y Singulares (ICTS), Valencia, Spain (A.T., L.M.-B.).; 4 Grupo de Nanobiotecnología para Ciencias de la Vida, Departamento de Química en Ciencias Farmaceuticas Facultad de Farmacia, Universidad Complutense de Madrid (UCM). Unidad de Microscopia e Imagen Dinamica, Centro Nacional de Investigaciones Cardiovasculares (CNIC), Centro de Investigación Biomédica en Red de Enfermedades Respiratorias (CIBERES), Instituto de Salud Carlos III (ISCIII), Madrid, Spain (K.O.-P., M.F.).; 5 QUIBIM SL – Quantitative Imaging Biomarkers in Medicine, Valencia, Spain (A.A.-B.).; 6 Departamento de Cirugía Experimental, Hospital Universitario La Paz, Madrid, Spain (C.L.-A.).; 7 Unidad de Fisiología, Departamento de Biología de Sistemas, Universidad de Alcalá (IRYCIS), Alcalá de Henares, Madrid, Spain (M. Saura).; 8 Centro de Investigación Biomédica en Red de Enfermedades Cardiovasculares (CIBERCV), Instituto de Salud Carlos III (ISCIII), Madrid, Spain (R.R.-C., M. Sanmartin, I.H., L.T., M. Saura, J.L.Z., C.Z.).

**Keywords:** extracellular matrix, myocardial infarction, nanotechnology, necrosis, reperfusion

## Abstract

Rapid screening and accurate diagnosis of acute myocardial infarction are critical to reduce the progression of myocardial necrosis, in which proteolytic degradation of myocardial extracellular matrix plays a major role. In previous studies, we found that targeting the extracellular matrix metalloprotease inducer (EMMPRIN) by injecting nanoparticles conjugated with the specific EMMPRIN-binding peptide AP9 significantly improved cardiac function in mice subjected to ischemia/reperfusion.

**Methods:**

In a porcine model of coronary ischemia/reperfusion, we tested the theragnostic effects of administering 0.1 mg/kg gadolinium-containing nanoparticles conjugated with AP9 (NAP9), a synthetic peptide that targets EMMPRIN or a control nanoparticle (NAPSC). Cardiac magnetic resonance assessment of the infarct progression, ventricular function, and nanoparticle distribution was performed the next 7 days. We also measured the infarcted area of the heart and cardiac remodeling at 7 or 21 days after ischemia/reperfusion.

**Results:**

After 21 days of ischemia/reperfusion, NAP9 reduced the extension of cardiac necrosis (14.1±9.7 versus 35.5±1.8) and the levels of collagenolytic activity of MMPs (matrix metalloproteases), along with a significant reduction in collagen deposition (7.5±4.5 versus 41.3±20); including the ratio of type I versus III collagen fibers in the necrotic myocardium. In terms of cardiac function, the response to NAP9 administration resulted in a significant improvement of cardiac performance overtime, as evidenced by the left ventricle ejection fraction (64.0±7.8), when compared with those present in the NAPSC group (47.3±4.7). As shown by magnetic resonance imaging, noninvasive molecular imaging of NAP9 enabled us to find a significant reduction in cardiac necrosis, myocardial edema, hemorrhage, and microvascular obstruction, suggesting that NAP9 may reduce myocardial injury and preserve left ventricular function, at least, by preventing the effect of EMMPRIN on extracellular matrix degradation.

**Conclusions:**

Our data point towards NAP9 as a promising theragnostic tool in managing acute myocardial infarction, by inhibiting EMMPRIN-induced extracellular matrix degradation and allowing noninvasive visualization of cardiac necrosis progression over time.

**Clinical Perspective**

- The use of nanotechnology applied to the generation of new molecular targets represents one of the greatest technological challenges in cardiovascular imaging, providing significant advantages that include a lack of undesired immune responses. Nanoparticles show theragnostic features combining both diagnostics and therapeutics properties in one single compound, and noninvasive molecular imaging may represent a significant breakthrough in the study of myocardial infarction enabling high-resolution tissue visualization but also featuring imaging detection of a target molecule over time. Such is the case of extracellular matrix metalloproteinase inducer, a potent activator of proteolytic degradation of the extracellular matrix which expression increases at the onset of myocardial ischemia, as one of the most powerful effectors of cardiac necrosis. By using gadolinium-containing nanoparticles conjugated with specific extracellular matrix metalloproteinase inducer-binding peptides, we delimited the myocardial ischemic area and inhibited myocardial fibrosis, myocardial extracellular volume, microvascular obstruction, intramyocardial hemorrhage, and edema, and at the same time, the nanoparticles inhibited extracellular matrix degradation, and hence myocardial necrosis, improving cardiac contractility. Our preclinical research in large animal models of myocardial infarction takes a further step towards technological development for a new recent upcoming diagnosis and treatment methods of acute myocardial infarction.

**See Editorial by Thackeray & Hess**

Early diagnosis and treatment are critical to reduce harmful outcomes in patients after acute myocardial infarction (AMI).^1^ In recent years, multiple therapeutic approaches have emerged, but currently, only a few reduce myocardial injury. Cardiac magnetic resonance imaging (MRI) is the noninvasive and most reliable procedure to study myocardial injury, and it provides valuable information in the search for new therapies.^2^

Targeting extracellular matrix (ECM) degradation is a promising therapeutic approach, given that ischemia-induced ECM proteolytic activation often leads to extensive cardiac cell necrosis, adverse ventricular remodeling, and heart failure.^3^ In this context, the ECM metalloproteinase inducer (EMMPRIN; CD147, Basigin), a glycoprotein that promotes MMP (matrix metalloprotease)-induced ECM degradation when is highly glycosylated EMMPRIN, is a potential target that plays a key role in the inflammatory response to cardiac ischemia, by regulating the expression of MMP-9 and MMP-13.^4–7^

To study whether EMMPRIN may serve as a therapeutic tool, many laboratories have targeted EMMPRIN through different approaches. We and others have used antibodies against EMMPRIN,^8,9^ mice deficient for genes involved in cardioprotection^10^ or, more recently, specific nanoparticles conjugated with the EMMPRIN-binding peptide AP9 in mice,^11^. However, no large animal studies have been reported so far of the potential benefit of using EMMPRIN as a therapeutic target in cardioprotection.

The use of nanoparticles has emerged for their theragnostic capacity against pathologies that include myocardial infarction,^11–13^ infectious diseases,^14^ central nervous system diseases,^15^ or even cancer.^16^ The rationale of using micelle or iron oxide-based nanoparticles lies on the significant advantages over other strategies. The use of nanoparticles tends to reduce in many cases the adverse effects associated with undesired immune responses, as when using antibodies, and in general, given their small size and chemical composition, they exhibit very low or no toxicity, as we previously reported in mice.^11^ Once more, testing in large animal models of disease is, in most cases, pending of investigation.

In a mouse model of AMI, we previously reported that nanoparticles conjugated with AP9 (NAP9), a nanoparticle conjugated with the specific EMMPRIN-binding peptide AP9,^11^ effectively improved the cardiac function of hearts subjected to ischemia/reperfusion (IR). In this work, we extend our study to a porcine model of coronary IR to evaluate its potential use in clinical practice.

We hypothesized that targeting EMMPRIN with NAP9 is a promising theragnostic tool by allowing noninvasive visualization of myocardial infarction while inhibiting ECM degradation and improving left ventricular (LV) function. We found that NAP9 reduces infarction size and locates preferentially in the infarcted tissue compared with pigs injected with NAPSC.

## Methods

The data that support the findings of this study are available from the corresponding author upon reasonable request.

All the surgical procedures were performed in the Experimental Surgery Department of the Hospital Universitario La Paz in Madrid and The Medical Research Institute Hospital La Fe (IIS La Fe) in Valencia, in accordance with the following institutional guidelines: Guide for the Care and Use of Laboratory Animals published by the US National Institutes of Health (Publication No. 85-23, revised 1985), and the Animal Welfare Ethics Committee and complied with the EU Directive on experimental animals (63/2010 EU) and related Spanish legislation (RD 53/2013). IRB approval was obtained (PROEX 138/17) to perform the animal procedures (no informed consent was required by the participants).

### Reagents and Equipment

Evans Blue, triphenyl tetrazolium chloride, hematoxylin-eosin, sirius red, Triton X-100, calcium chloride, zinc chloride, and acetic acid were from Merck (Madrid, Spain). The Masson trichrome staining kit, anti-EMMPRIN, anti-CD68, HRP/DAB IHC kit, and HRP-conjugated secondary antibodies were from Abcam (Cambridge, United Kingdom). Anti-MMP-9 and anti-MMP-13 were from Santa Cruz (Heidelberg, Germany). Ketamine was from Pfizer (New York, NY); the isoflurane was from Abbvie (North Chicago, IL); the propofol was from Fresenius (Bad Homburg, Germany); the fentanyl was from Kern Pharma (Madrid, Spain); the diazepam was from Roche (Basel, Switzerland); and the amiodarone was from Sanofi Aventis (Gentilly, France).

### Animal Model of Coronary IR

All the surgical procedures were performed in the Experimental Surgery Department of the Hospital Universitario La Paz in Madrid and The Medical Research Institute Hospital La Fe (IIS La Fe) in Valencia, in conforming to the Guide for the Care and Use of Laboratory Animals published by the US National Institutes of Health (Publication No. 85-23, revised 1985), and the Animal Welfare Ethics Committee and complied with the EU Directive on experimental animals (63/2010 EU) and related Spanish legislation (RD 53/2013). PROEX 138/17.

Twenty-eight Yorkshire pigs (36±5 kg) were housed 1 week before surgery to avoid unease or stress associated with the new environment. Before the surgical intervention, animals were anesthetized with intramuscular ketamine 10 mg/kg and midazolam 0.5 mg/kg. Anesthesia was induced by inhaled isoflurane and maintained with continuous infusion of propofol 2 mL/kg per hour, fentanyl 50 µg/kg per hour, and diazepam 10 µg/kg per hour. After intubation and mechanical ventilation with 100% oxygen saturation, 5000 IU of heparin and amiodarone 2 mg/kg per hour were administered to avoid blood clotting of catheters and malignant cardiac arrhythmias, respectively. Before complete occlusion, the hearts were subjected to ischemic preconditioning by blocking the left anterior descending artery for short periods (1, 3, and 5 minutes each). IR was produced by occluding left anterior descending artery for 45 minutes using a JL3 6F catheter and an angioplasty balloon. The complete myocardial ischemia was confirmed by ST-segment elevation. The balloon was then removed to reopen the artery, and animals received Placebo (physiological saline solution), NAP9, or NAPSC (0.1 mg/kg; see Supplemental Methods for nanoparticle cytotoxicity assay), prepared as previously described.^11^ After 7 days of reperfusion, animals were euthanized to extract the heart. Immunoblot samples were immediately frozen at −80 °C, and histology samples were included in 4% Formalin.

### Cardiac Magnetic Resonance

Images were acquired on a 3 Tesla MR scanner (Philips Achieva 3.0 TX) with multi-transmission technology, using a 32-channel XL Torso coil specifically for human torso acquisitions. Acquisitions were made before ischemia, basal cardiac magnetic resonance (CMR), and immediately after reperfusion. Nanoparticles were injected, and postinjection acquisition was performed at times 0-hour, 24-hour, 72-hour, and 7-day postinjection. The number of animals subjected to MR were 8 (4 per group).

To estimate LV function, contiguous images were acquired covering the LV from the base to the apex with a parallel imaging Balanced Turbo Fast Field Echo Multi-Slice sequence, with a 45° flip angle, 2.9 ms repetition time and 1.4 ms echo time. The field of view was 280×280 mm with a resolution of 1.8 mm in plane and 6 mm in slice thickness.

The quantification of edema as the area at risk (AAR) was performed on a T2-weighted short-TI inversion recovery turbo fast spin-echo sequence (repetition time=1429 ms and echo time=80 ms).

Myocardium characterization was extended with the acquisition of a Multi-Eco sequence Multi-slice mFFE sequence for the study evaluation of the transverse relaxation time (T2*) and ratio (R2*). The precontrast and postcontrast Modified Look-locker Imaging sequences allowed us to obtain the longitudinal relaxation time (T1) and calculate the extracellular volume (ECV).

The contrast delayed enhancement was evaluated after the administration of gadolinium (0.2 mmol/kg), applying an inversion recovery/fast echo gradient sequence (repetition time=8 ms; echo time=4 ms; field of view=300×225; matrix=256×144; and slice thickness=6 mm).

The LV ejection fraction (LVEF) was obtained by delimiting the perimeter of the endocardium and epicardium at the moments of systole and diastole on the balanced turbo fast field echo sequence.

The volume of regional edema and myocardium at risk was quantified after its detection by semi-automatic region recognition algorithms, based on thresholds and contour detection on the T2-weighted short-TI inversion recovery turbo fast spin-echo sequence.

The transverse relaxation time (T2*) was calculated voxel-by-voxel from the different described regions to obtain the T2* and R2* maps over the areas of interest. The calculation of the transverse relaxation times was made as follows:

$$I_{t}=\;I_{t=0\;}*\;{\text{e}}^{-t/T2^{*}}\;T2^{*}=\;\frac{-\Delta \text{TE}}{\text{ln}(\frac{I_{TE2}}{I_{TE1}})}$$

We obtained the T1 relaxation time calculation from the Modified Look-locker Imaging sequence as a T1 map for each voxel of the myocardium, which will serve as potential additional information for detecting the myocardial AAR and fibrosis.

The edema volume and myocardial AAR were quantified through semi-automatic algorithms for region recognition, based on thresholds and contour detection on the delayed enhancement images. The ECV map was obtained to get quantitative information about the state of the myocardium to estimate viability on each segment.

### Echocardiography

Echocardiography was performed to determine LV function using a Vivid Q ultrasound system from GE Healthcare (General Electric, Chicago, IL), equipped with a 1.9-4 MHz scan head. Parasternal long and short-axis-view images of the heart were taken before the surgery, at the end of ischemia, and at the end point to determine LV function worsening and recovery. The parameters studied using the on-site software cardiac package were: Diastolic interventricular septum thickness, systolic and diastolic left ventricle internal diameter, systolic and diastolic left ventricle posterior wall thickness, LVEF, LV shortening fraction, heart rate, cardiac output, and LVEF. The same operator performed data acquisition and analysis to avoid the interobserver error.

### Histological Procedures

The necrotic area of the hearts was measured by Evans Blue/triphenyl tetrazolium chloride staining. The left anterior descending artery was reoccluded in the same location as day 0. Then, 200 mL of 5% Evans Blue solution was injected using a 5F fenestrated Pigtail catheter within the LV to distribute the compound across the entire cardiovascular system, excepting the blood-deprived area of the heart. After 2 minutes, the animal was sacrificed, and the heart extracted and frozen at −20 °C for 24 hours. Eight pigs (4 per group) were euthanized 7 days after the surgery and 8 pigs (4 per group) were euthanized 21 days following the same method described before. The next day, hearts were cut into 0.8 cm slices and incubated in 1% triphenyl tetrazolium chloride for 20 minutes at 37 °C and next in 10% paraformaldehyde solution. The necrotic area was relativized to the AAR to avoid interexperiment variability concerning the ischemic area.

Heart morphology (10 pigs; 5 per group) was visualized by hematoxylin/eosin staining, and Masson trichrome staining detected collagen deposition. Collagen fibers were stained with sirius red for 1 hour and washed using acetic acid. Total collagen was assessed under bright-field microscopy and type I and III collagen fibers were visualized and differentiated under normal and polarized light.

CD68 expression was detected using the Mouse and Rabbit HRP/DAB Detection IHC kit (ab64264) from abcam following the manufacturer’s instructions.

### Immunoblotting

Proteins from 10 hearts (5 per group) were extracted to measure EMMPRIN, MMP-9, and MMP-13 expression. Twenty micrograms of total protein were loaded into 10% polyacrylamide gels. After the electrophoresis, proteins were transferred to polyvinylidene difluoride membranes and blocked with 5% BSA in T-TBS. Membranes were incubated for 1 hour with anti-EMMPRIN 1:500 and anti-Rabbit HRP-conjugated secondary antibody. Then, protein bands were visualized by chemiluminescence and studied using image analysis software.

### Gel Zymography

Protein lysates prepared from 10 hearts (5 per group) were resolved on 7.5 % SDS-polyacrylamide gels containing 1 mg/mL gelatin. Gels were incubated for 30 minutes with 2.5% Triton X-100 buffer, and then incubated over 16 hours with developing buffer (50 mmol/L Tris-HCl, pH 7.5, 200 μmol/L NaCl, 10 μmol/L CaCl2, 0.02% Brj35). Gels were stained with Coomassie blue for 30 minutes, and gelatinolytic activity was visualized after incubation with unstaining solution (methanol:acetic acid:water, 50:10:4). Optical densities were analyzed using image analysis software and collagenolytic activity was measured by analyzing the active/zymogen ratio.

### Statistical Analysis

To estimate the sample size for each group we performed a comparative study of 2 independent means from similar studies, assuming a pooled SD of 5 units. Therefore, we projected a sample of 4 pigs per group (N=8) to achieve a power of 80% and a level of significance of 5% (2 sided), for detecting a true difference in means between the test and the reference group of 10 (ie, 65–55) units.

Unless otherwise specified, data are expressed as mean±SD. The samples were normally distributed, and homogeneity of variances between groups was verified by performing a Levene test. After confirming equal variance across samples, mean values between study groups were compared by using a Wilcoxon rank-sum test. Differences were considered significant at *P*<0.05. Error bars represent±SD. The number of animals used in each experiment is specified in the text.

To further map the distribution of nanoparticles within the LV, we used MRI to study NAP9 and NAPSC relaxation rates (ΔR1) in each segment of the LV, following the American Heart Association 17-segment model. We used the Spearman rank correlation to calculate the correlation between native T1 relaxation times and NAP9 ΔR1 for each segment at days 1, 3, and 7 after IR, analyzing the presence of 5 different points/pig, located in the following segments:

Point 1: anterior/septal location (segments 7 and 8). Point 2: inferior/septal location (segments 9 and 10). Point 3: lateral location (segments 11 and 12). Point 4: anterior/septal location (segments 13 and 14). Point 5: inferior/lateral location (segments 15 and 16). These points were defined to discriminate the location of the ischemic tissue in the 4 walls of the heart (anterior, lateral, basal, or septal).

## Results

### NAP9 Reduces Scar Formation and Infarct Size After 7 Days of IR

**Figure 1.**
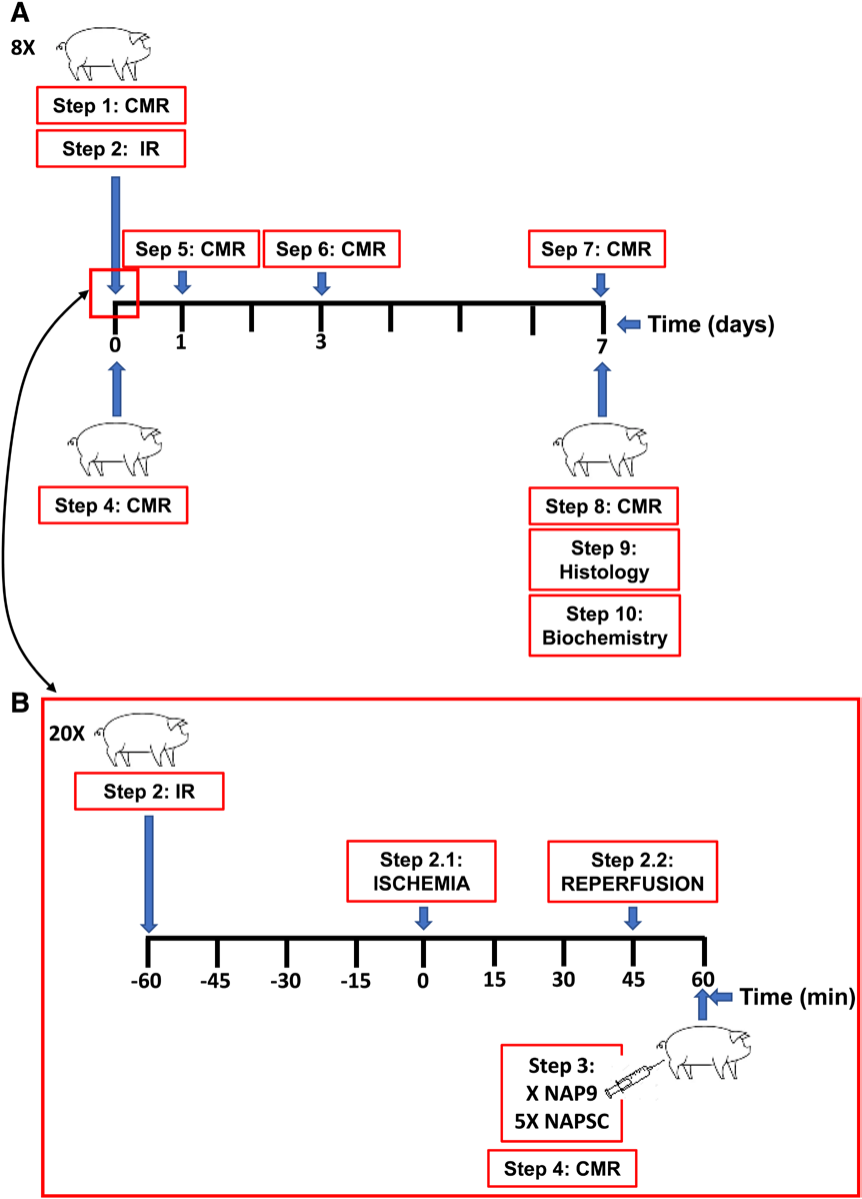
**Schematic representation of the study. A**, Procedures depicted over 7 d and (**B**) procedures performed within the 2 h. CMR indicates cardiac magnetic resonance; IR, ischemia/reperfusion; and NAP9, nanoparticles conjugated with AP9.

**Figure 2.**
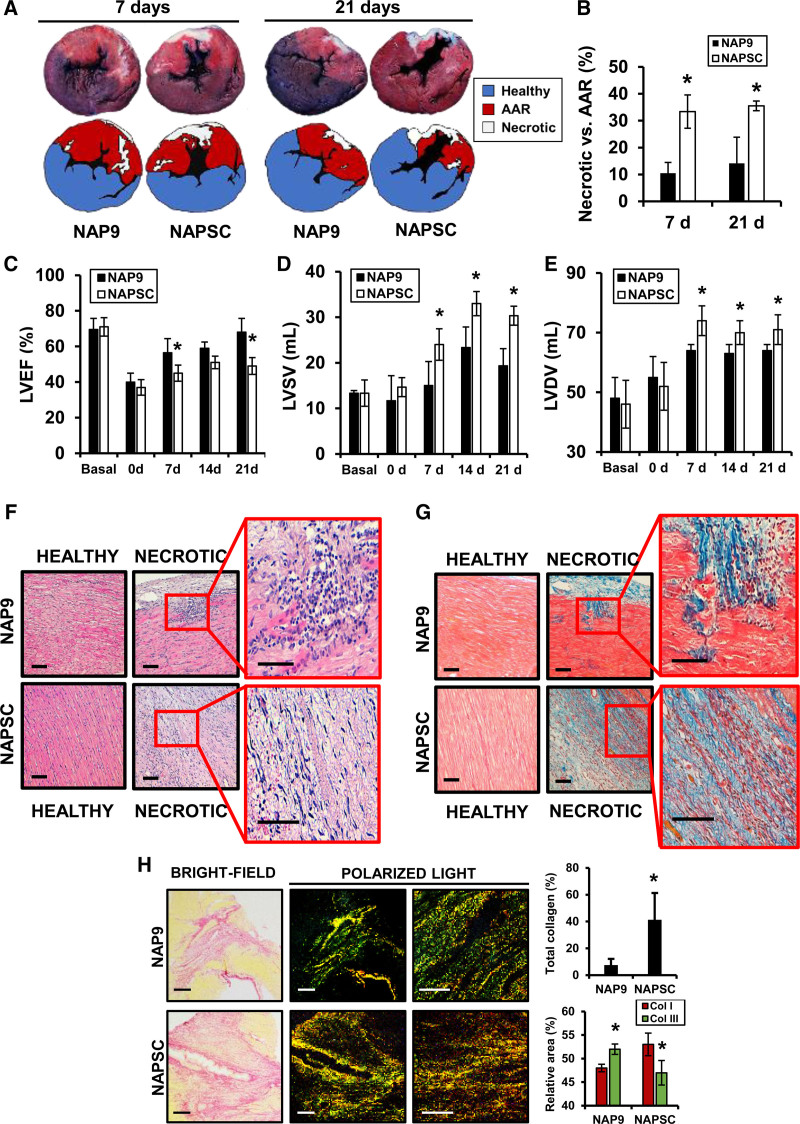
**Nanoparticles conjugated with AP9 (NAP9) reduces scar formation and infarct size and preserves ventricular function. A** and **B**, Evans blue/triphenyl tetrazolium chloride (TTC) double staining of 0.8 cm heart sections isolated 7 and 21 d after ischemia/reperfusion (IR) from pigs treated with 0.1 mg/kg NAP9 or NAPSC. N=8 pigs/group (*mean±SD; *P*<0.05; NAP9 vs NAPSC); (**C**) left ventricle ejection fraction (LVEF); (**D**) left ventricle end systolic volume (LVSV); (**E**) left ventricle end diastolic volume (LVDV) from the same hearts as in **A**, before (pre), after IR (post), 7, 14, and 21 d post-IR. N=8 pigs/group (*mean±SD; *P*<0.05; NAP9 vs NAPSC); (**F**) hematoxylin-eosin (H/E) staining of heart sections from healthy and necrotic areas of hearts collected from pigs treated with 0.1 mg/kg NAP9 or NAPSC (blue=nuclei; pink=cytoplasm. N=5 pigs/group); (**G**) Masson trichrome staining of healthy and necrotic sections (red=muscle fibers; blue=collagen; brown=nuclei; N=5 pigs/group); (**H**) Sirius red staining of necrotic heart sections (bright-field: yellow=muscle fibers; red=collagen. Polarized light: red/yellow=type I collagen; green=type III collagen). N=3 pigs/group (*mean±SD; *P*<0.05 NAP9 Col I vs d Col III; *P*<0.05 NAPSC Col I vs Col III). AAR indicates area at risk.

Animals were subjected to coronary IR and treated with 0.1 mg/kg NAP9 or NAPSC as depicted (Figure 1). We calculated the percentage of necrotic myocardium referenced to the AAR by day 7 and 21 after IR in 0.8 cm heart sections stained with Evans Blue/triphenyl tetrazolium chloride. The necrotic areas were significantly reduced in NAP9 treated animals (Figure 2A and 2B) by day 7 (10.5±4.1 versus 33.4±6.2) and 21 (14.1±9.7 versus 35.5±1.8), resulting in improved cardiac function, as seen by the ejection fraction, the LV systolic volume values and LV diastolic volume values after 21 days of IR (Figure 2C through 2E). Infarcted area, LVEF, LV systolic volume, and LV diastolic volume values (mean±SD) can be found in the Table.

**Table.**
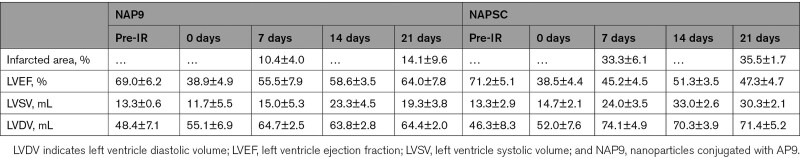
Necrotic Areas in the Myocardium (% Respect to Area at Risk) and Mean LVEF (%), LVSV (mL), and LVDV (mL) Values

In addition, cardiac structure was assayed by hematoxylin-eosin staining of heart sections by 7 days after reperfusion, noticing that in pigs treated with 0.1 mg/kg NAP9 the ventricular morphology was preserved (Figure 2F). While the amount of inflammatory and necrotic cell foci was significantly increased (Figure S2A), as well as inflammatory macrophages present in the group of NAPSC (Figure S2B). NAP9 also decreased collagen deposition as detected by Masson trichrome staining of the same sections (Figure 2G), in particular the ratio of type I versus type III collagen fibers in the necrotic myocardium, as detected by sirius red staining of heart sections and visualized with polarized light (Figure 2H).

### NAP9 Reduces Myocardial Injury and Preserves LV Function

**Figure 3.**
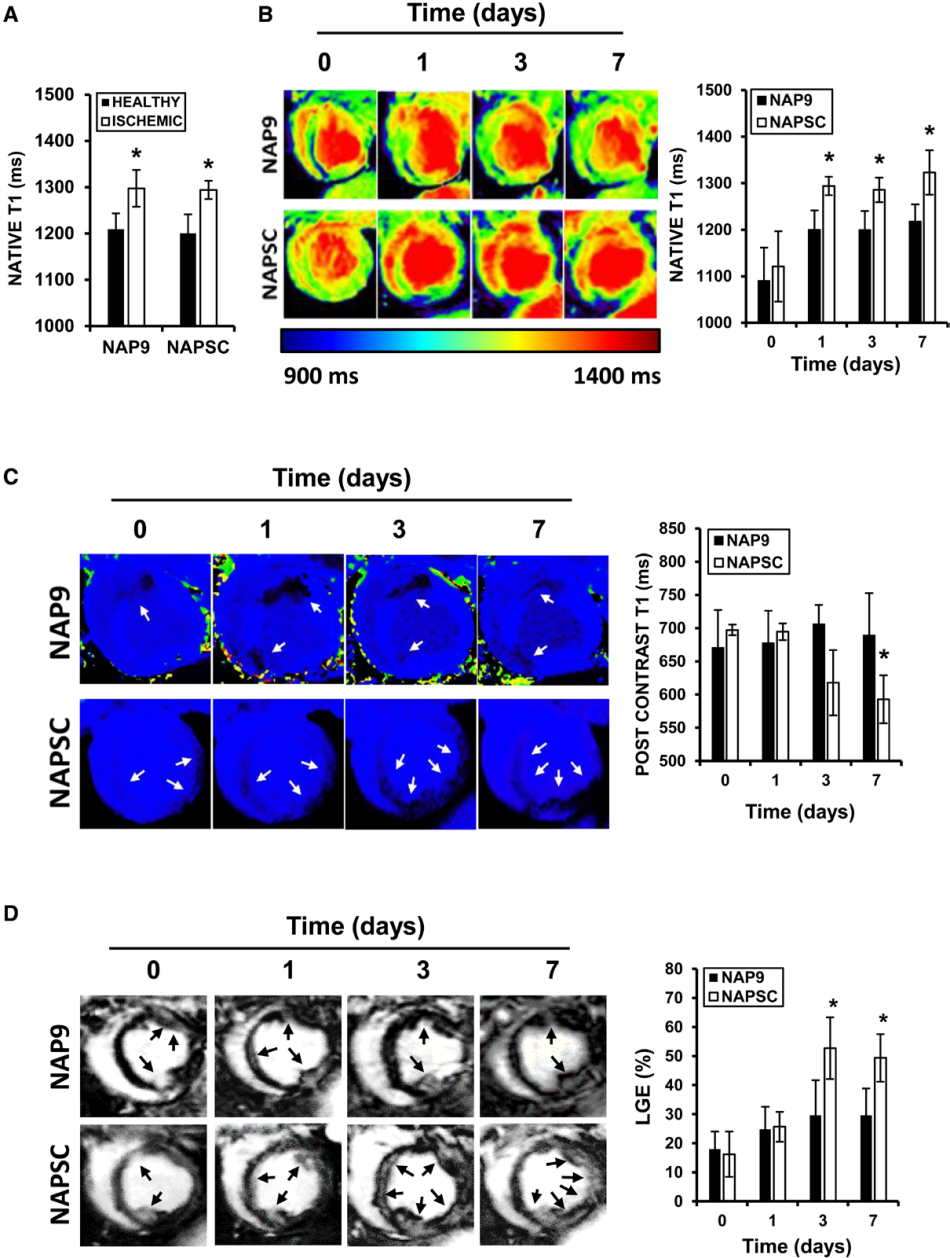
**Nanoparticles conjugated with AP9 (NAP9) reduces myocardial injury and infarct progression. A**, Native T1 relaxation times of healthy and ischemic hearts from pigs injected with NAP9 or NAPSC 24 h after ischemia/reperfusion (IR). N=4 pigs/group (*mean±SD; *P*<0.05; healthy vs ischemic). **B**, Colored native T1 maps of hearts from pigs injected with NAP9 or NAPSC and relaxation times of the ischemic area. N=4 pigs/group (*mean±SD; *P*<0.05; NAP9 7 d vs NAPSC). **C**, Colored postcontrast T1 maps of hearts from pigs injected with NAP9 or NAPSC and relaxation times of the ischemic area. N=4 pigs/group (*mean±SD; *P*<0.05; NAP9 7 d vs NAPSC). **D**, Late gadolinium enhancement (LGE) detected at the times indicated. N=4 pigs/group (*mean±SD; *P*<0.05; NAP9 vs NAPSC).

Myocardial injury was also evaluated by MRI, analyzing the MRI-T1 and the -T1 Postcontrast relaxation times. First, we determined the AAR by delimiting the ischemic area (native T1 relaxation times >1250 ms) 24-hour post-IR (Figure 3A). Subsequently, we analyzed the native and postcontrast T1 maps by day 3 and 7 post-IR to assess myocardial injury progression. We observed that NAP9 effectively decreased native T1 relaxation times by day 7 post-IR (Figure 3B) and preserved T1 postcontrast values in the ischemic myocardium, while at the same time, the group of NAPSC showed a significant decrease at 3- and 7-day post-IR (Figure 3C). We analyzed whether there was evidence of late gadolinium enhancement and the percentage of affected myocardium to visualize regional scar formation and myocardial fibrosis. We found a decrease of late gadolinium enhancement in the myocardium area of pigs injected with NAP9 by day 7 after IR, when compared with the group of NAPSC (Figure 3D), suggesting that administration of NAP9 significantly delays the progression of myocardial necrosis overtime after IR.

### NAP9 Inhibits the Expression of EMMPRIN in Response to IR

**Figure 4.**
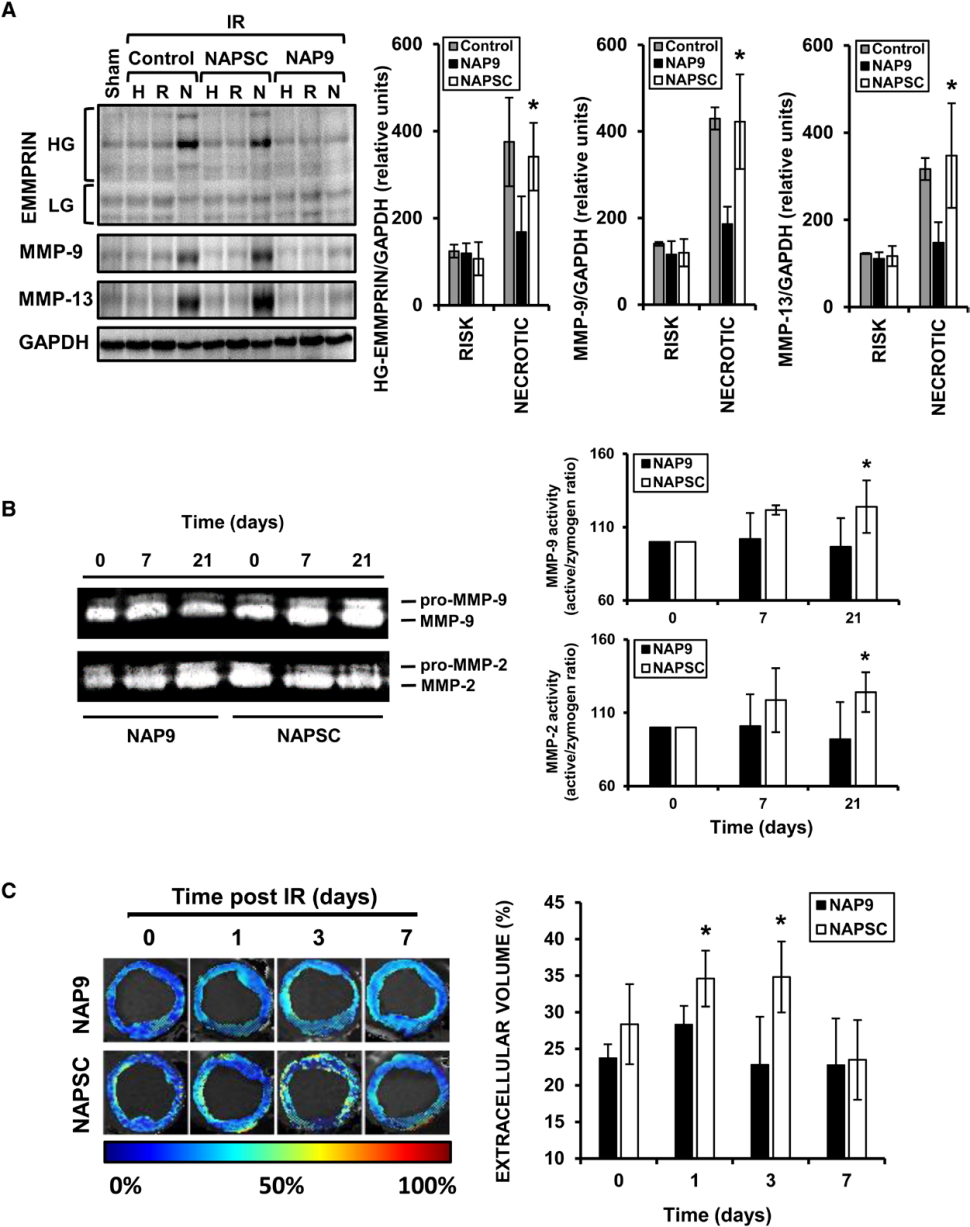
**Nanoparticles conjugated with AP9 (NAP9) reduces extracellular matrix metalloproteinase inducer (EMMPRIN)-related myocardial remodeling. A**, Immunoblot detection of EMMPRIN, MMP (matrix metalloprotease)-9, and MMP-13 in healthy (H), at risk (R), and necrotic (N) sections of hearts from pigs treated with 0.1 mg/kg NAP9, NAPSC, or control (physiological saline solution). N=5 pigs/group (*mean±SD; *P*<0.05; NAP9 vs NAPSC). **B**, MMP-2 and MMP-9 gelatinolytic activity present in the hearts of pigs injected with 0.1 mg/kg NAP9 or NAPSC after 0, 7, and 21 d of ischemia/reperfusion (IR). N=5 pigs/group (*mean±SD; *P*<0.05; NAP9 vs NAPSC). **C**, Extracellular volume (ECV) of the ischemic zone of hearts from pigs treated with NAP9 or NAPSC. N=4 pigs/group (*mean±SD; *P*<0.05; NAP9 vs NAPSC). HG EMMPRIN indicates high-glycosylated EMMPRIN.

To test whether in pigs NAP9 may also prevent proteolysis, we injected 0.1 mg/kg NAP9 or NAPSC into pigs subjected to IR, finding that in the necrotic area, a significant reduction in high (highly glycosylated: active EMMPRIN) and low glycosylated EMMPRIN, MMP-9, and MMP-13, were detected by immunoblot with protein extracts isolated after 7 days of IR (Figure 4A). Interestingly, MMP-2 and MMP-9 gelatinolytic activity was highest at day in the NAPSC treated pigs (Figure 4B), suggesting that NAP9-induced cardiac protection may be a result of, at least, the prevention of ECM degradation.

We also analyzed myocardial ECV by MRI, as a matter to quantification of cardiac remodeling, finding that administration of 0.1 mg/kg NAP9 effectively prevented the increase of myocardial ECV post-IR. Simultaneously, hearts from the NAPSC group also showed a significant increase at 1- and 3-day post-IR (Figure 4C).

### NAP9 Reduces Microvascular Obstruction, Intramyocardial Hemorrhage, and Edema After 7 Days of IR

**Figure 5.**
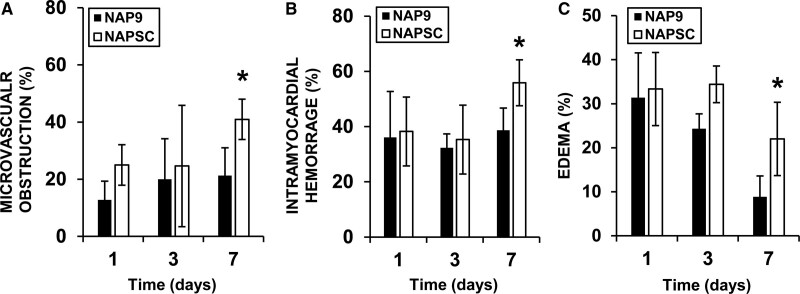
**Nanoparticles conjugated with AP9 (NAP9) reduces microvascular obstruction, intramyocardial hemorrhage, and edema. A**, Percentage of myocardium affected by microvascular obstruction; (**B**) intramyocardial hemorrhage (IMH); and (**C**) edema. N=4 pigs/group (*mean±SD; *P*<0.05; NAP9 vs NAPSC).

Following coronary IR, the risk of experiencing major adverse cardiovascular events is increased, and the presence of microvascular obstruction (MVO), intramyocardial hemorrhage (IMH), and edema are strong predictors of major adverse cardiovascular event. To determine whether NAP9 might reduce reperfusion-mediated major adverse cardiovascular event, we analyzed native T1, T2, and T2* values to address the presence of MVO (native T1 1150–1250 ms and T2* <20 ms), IMH (T2* <20 ms), and edema (T2 >70 ms). Cardiac IR induced a 20% increase of MVO (Figure 5A), but administration of 0.1 mg/kg NAP9 prevented a further increase by day 7 after IR, whereas NAPSC did not have any effect, and the same was observed for IMH (Figure 5B). Interestingly, the impact of NAP9 in the reduction of cardiac edema was maximal after 7 days of IR (Figure 5C).

### NAP9 Shows Specific Tropism Towards Infarcted Myocardium

We previously found that binding NAP9 to EMMPRIN allowed us to noninvasively visualize cardiac necrosis in mice subjected to IR. Here, by using postcontrast T1 and analyzing myocardial relaxation rates (ΔR1), a more significant acquisition of NAP9 was found in hyperintense T1 sections.

To determine the distribution of the nanoparticles within the LV, we studied NAP9 and NAPSC relaxation rates (ΔR1) in each section of the LV, following the American Heart Association 17-segment model. We represented native T1 values and nanoparticles ΔR1 following a color code of 7 to 16 to compare native T1 hyperintense and the nanoparticles enriched areas. Gray-colored graphics represent native T1 values of the LV corresponding lighter tones to hypointense sections (<1200 ms) becoming darker, while the signal increase (1200–1225; 1225–1250; >1250 ms). Concerning nanoparticles ΔR1, NAP9 graphics were depicted in blue, and NAPSC was colored in red. Lighter tones correspond to low ΔR1 (<4 1/s) and darker tones to higher ΔR1 (4–5; 5–6; and >6 1/s). Graphics show results from days 1, 3, and 7 since data from day 0 showed no significant effects (Figure S3).

Twenty-four hours after AMI, we observed that after the injection of 0.1 mg/kg of NAP9, specific LV segments showed higher ΔR1 than others. Figure S3A is a representative LV graphic showing the distribution of NAP9, in which we discriminated the specific segments corresponding to NAP9 accumulation, and interestingly, segments with higher ΔR1 also presented a hyperintense native T1 signal. Figure S3B and S3C show the same heart after the injection of 0.1 mg/kg of NAP9 at 3 and 7 days after surgery. We observed that NAP9 not only preferentially aggregated in almost the same areas as day 0, indicating tropism specificity, but also we found that segments with higher ΔR1 corresponded with those with higher native T1 signal, suggesting that NAP9 aggregates specifically in hyperintense T1 segments, thus, in the necrotic sections. Also, NAP9 showed specific tropism to these zones on different days, indicating its reliability to study myocardial infarction progression.

**Figure 6.**
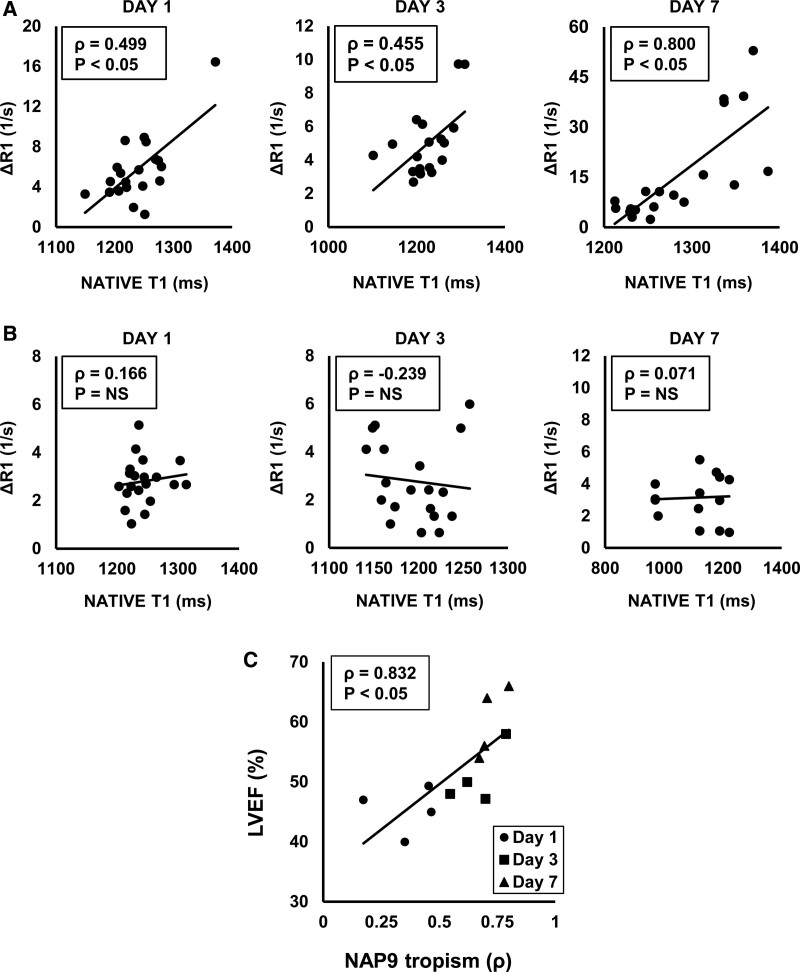
**Nanoparticles conjugated with AP9 (NAP9) shows specific tropism towards infarcted myocardium. A**, Scatterplot shows the correlation of native T1 relaxation times and ΔR1 of hearts from animals treated with 0.1 mg/kg NAP9 at 1, 3, and 7 d post-acute myocardial infarction (AMI). N=4 NAP9. **B**, Scatterplot shows the correlation of native T1 relaxation times and ΔR1 of hearts from animals treated with 0.1 mg/kg NAPSC at 1, 3, and 7 d post-AMI. N=4 NAPSC. **C**, Scatterplot shows the correlation between left ventricle ejection fraction (LVEF) and NAP9 aggregation in the necrotic area (as measured by Spearman rank correlation between ΔR1 vs native T1) of hearts from animals treated with 0.1 mg/kg NAP9, and measured by days 1, 3, and 7 post-ischemia/reperfusion (IR). N=4 NAP9. ρ=Spearman rank correlation. NS indicates nonsignificant.

After injection of NAPSC, we noticed a nonspecific distribution within the LV at 1-, 3-, and 7-day post-IR. By 24 hours of IR, no significant aggregation was detected, whereas hyperintense T1 segments were corresponding to the necrotic area (Figure S3D through S3F), suggesting that accumulation of NAPSC could be at least partly explained by IMH or edema. To verify these results, we studied the correlation between native T1 relaxation times (*x* axis) and NAP9 ΔR1 (*y* axis) for each segment (Figure 6). A strong positive correlation was found by days 1, 3, and 7 after IR, confirming that NAP9 accumulates in T1 hyperintense segments of the LV (Figure 6A), while in the NAPSC group, we found no correlation between the segment’s native T1 signal (*x* axis) and NAPSC ΔR1, indicating that NAPSC aggregation was not specific in T1 hyperintense segments (Figure 6B).

To determine whether NAP9 tropism was related to an improved cardiac function, we analyzed the relationship between NAP9 accumulation in the myocardium and the ventricular function measured by LVEF after IR. NAP9 tropism (*x* axis) was defined for each animal by days 1, 3, and 7 by the Spearman rank correlation (ρ) between NAP9 relaxivity versus native T1 relaxation times. LVEF (*y* axis) of the same hearts was measured by day 1, 3, and 7 (N=4; Figure 6C).

## Discussion

In the current study, we tested in a porcine model of IR the use of gadolinium-containing paramagnetic lipidic nanoparticles conjugated with AP9, a synthetic peptide which specifically binds to EMMPRIN, allowing noninvasive visualization by molecular CMR. For the first time, we report that administration of NAP9 induce a cardioprotective effect in pigs beyond 7 days after AMI, as we previously described in mice and pigs,^11,17^ by reducing the necrotic area and improving LV function after 21-day post-IR. This is consistent with the concept of using EMMPRIN as a therapeutic target^8–10^ towards the early steps of acute heart failure post-IR.

Paramagnetic nanoparticles that modulate biological processes with high molecular specificity and affinity, due, in part, to their capacity to pass through the plasma membrane and tissue barriers, has emerged as potential agents in the treatment and diagnosis of cardiovascular disease.^18^ Current research of theragnostic nanoparticles in cardiovascular disease is at an early stage. However, several promising research lines are being developed nowadays, including studies that propose nanoparticles as drug delivery vehicles,^19^ to improve therapeutic cell effectiveness,^20^ or nanoparticles that target inflammatory-related pathways^18,21^ following AMI.

During AMI, the balance between ECM synthesis and proteolytic degradation is critical for disease progression. Abnormalities during this process may lead to aberrant crosslinking or adverse ratios between type I/III collagen matrix components,^22^ and hence, targeting MMP-induced ECM degradation may result as a promising strategy as adverse cardiac remodeling may result in LV dilation and enlargement of the collagen scar.^23^ Besides pharmacological inhibition of MMPs,^24^ in recent years, we and others focused our attention on the MMP inducer EMMPRIN as a target against progression of AMI, showing the benefits of EMMPRIN inhibition in the short-term post-AMI.^8,11,25^ Here, we found that NAP9-induced inhibition of EMMPRIN is a new tool against cardiac adverse effects even 3 weeks after IR.

After AMI, a poor disease prognosis will depend on how the heart responds in terms of ECM remodeling, and EMMPRIN plays a key role in this process,^26^ to such an extent that moderate levels of ECM degradation are often associated to cardiac repair and regeneration.^27^ Total collagen content, including the ratios between type I and III collagens are critical parameters in the resolution of AMI, as an increased proportion of type I collagen is related to a poor prognosis.^22^ Here, we found that NAP9 administration was correlated to a decrease on type I collagen deposition, suggesting that at least NAP9-induced cardiac protection may result as affecting at the collagen composition in the heart.

CMR is the most reliable noninvasive procedure for the diagnosis of several cardiovascular diseases, and noninvasive molecular imaging by CMR is a new promising tool. The main goal of EMMPRIN inhibition is to prevent adverse ECM degradation and myocardial remodeling and besides our former contributions on targeting ECM by CMR,^11,28^ here we use this imaging technology to target ECM degradation in the context of AMI, a new promising alternative and a consistent strategy using theragnostic compounds.

Native T1, postcontrast T1 mapping, and late gadolinium enhancement are commonly used in the diagnosis of myocardial injury post-AMI. Native T1 values increase with cell death, local edema, and hemorrhage,^29^ and in postcontrast T1 structural abnormalities and tissue damage can be detected.^30^ Likewise, late gadolinium enhancement is strongly related to tissue damage, but its reliability as a prognostic parameter of irreversible injury in AMI is still controversial.^31^ By using these parameters, we characterized NAP9-induced effect in delimiting the healthy area and AAR of the heart, analyzing the progression of myocardial injury. The results showed a significant decrease of the necrotic area by day 7 after IR, in accordance with the improvement of cardiac function as compared with the group of NAPSC.

Recently, Sado et al^31^ described that an increase of myocardial ECV in the onset of myocardial infarction, could be related to collagen deposition and fibrosis, and after that, ECV measurement have become a reliable marker of cardiac remodeling, and strongly related to adverse AMI resolution.^32,33^ Therefore, we studied this parameter after injection of NAP9, describing that treatment prevented ECV increase at days 1 and 3, but no differences were found between groups by day 7 after IR. This result implies that EMMPRIN inhibition effectively prevents early myocardial remodeling, as this effect is consistent with the activity peak of some EMMPRIN-activated MMPs (MMP-9 and MMP-2),^34^ suggesting that NAP9 could prevent the early induction of MMPs during the inflammatory response.

IMH and edema associated to AMI can be detected in T2 sequences thanks to deoxyhemoglobin paramagnetic properties produced after blood degradation and the increase in water content at the edematous location,^35,36^ together with MVO as a recurrent sequel after AMI, a strong predictor of adverse cardiac function and remodeling.^36^ Administration of NAP9 successfully prevented IMH, edema, and MVO, although we are not certain about the exact mechanism underlying this phenomenon. However, the inflammatory process is strongly related to these events, and EMMPRIN is the natural ligand of cyclophilin A, a key inflammatory element in the heart that partakes in microvascular injury as Jin et al^26^ demonstrated in a mouse model of ischemic stroke. In addition, previously we also found that nitric oxide prevents overexpression of EMMPRIN in a murine model of AMI^9^ and during carotid atherosclerosis, and therefore MVO after endothelial dysfunction, which is related to a depletion of NO availability, may be prevented after inhibition of EMMPRIN.^28^ However, further studies must be performed to explore role of NAP9 in these contexts.

We studied whether NAP9 could be detected in CMR postcontrast T1 maps, as it contains gadolinium as a principal component, but unexpectedly, and although we previously found the rhodamine containing NAP9 by confocal microscopy in the heart,^17^ there was no significant CMR signal detected in the hearts of NAP9 treated animals, which could be explained by a low concentration of gadolinium-containing NAP9. Thus, we explored alternative approaches, starting with CMR sequences to detect myocardial relaxation rates (ΔR1), which are relative to the nanoparticle concentration.^37^ We detected that NAP9, but not NAPSC, specifically aggregates in the necrotic tissue, providing valuable evidence about the diagnostic reliability of using NAP9, and allowing in vivo visualization and measurement of the myocardial infarction. This is of significant interest, since as some authors point, heterogeneity between patients is a main problem in the development of new therapies.^27^ Hence, precise measurement tools may help to avoid these biasing, thus improving precise diagnosis and forecast progression of disease.

Limitations of the study may include the reduced sample size affecting at the clinical translation of results, which is related to the budget required to perform these studies. Another limitation is the animal model itself, since a porcine procedure o IR is, for several reasons, the best experimental approach.

## Conclusions

Noninvasive targeting of ECM remodeling is a reliable strategy and, to our knowledge, this is the first time in which a theragnostic tool is used in this context. Our results open a new range of possibilities for the treatment of AMI, as aiming ECM degradation and cardiac remodeling is not only a reliable approach to measure and visualize infarction but also this approach is designed to induce cardiac protection as well. Although further studies must be done to deeply understand NAP9 contribution, we show strong evidence of functionality to consider using NAP9 in future clinical studies as a new treatment against AMI.

## Article Information

### Sources of Funding

This work was supported by C. Zaragoza: Universidad Francisco de Vitoria (grant numbers 2017/18), Fundación BBVA Ayudas a Equipos de Investigación científica (2017), and Proyectos de i+D+I, from the program Investigación orientada a los retos de la sociedad, cofounded by Fondo Europeo de Desarrollo Regional (FEDER) A way to achieve Europe (MINECO/AEI/FEDER/EU SAF2017-87342-R), Dr Saura: Ayudas para realización de proyectos de investigación Junta de Comunidades de Castilla La Mancha SBPLY/19/180501/000055, cofounded by Fondo Europeo de Desarrollo Regional (FEDER). A way to achieve Europe. M. Filice: Comunidad Autonoma de Madrid and Universidad Complutense de Madrid for research project No.2017-T1/BIO-4992 Accion de Atracción de Talento.

### Disclosures

None.

### Supplemental Material

Supplemental Methods

Figures S1–S3

## Supplementary Material
