# Supplementary material for: Theranostic Contribution of Extracellular Matrix Metalloprotease Inducer-Paramagnetic Nanoparticles Against Acute Myocardial Infarction in a Pig Model of Coronary Ischemia-Reperfusion
Source: Circ Cardiovasc Imaging. 2022 Jun 9;15(6):e013379. doi: 10.1161/CIRCIMAGING.121.013379 (PMC9213084; doi:10.1161/CIRCIMAGING.121.013379)
Supplement: Supplementary file 1 [file hci-15-e013379-s001.pdf]

SUPPLEMENTAL MATERIAL

Supplemental Methods

Nanoparticle cytotoxicity

Cytotoxicity was performed by intravenous injection of 0, 0.01, and 0.1 mg/kg NAP9 in pigs, measuring serum concentrations of aspartate transaminase (AST), alanine transaminase (ALT), creatinine (CREA), and creatine kinase MB (CK-MB) as markers of hepatic injury (AST, ALT), renal injury (CRE), and cardiac necrosis (CK-MB). A dose of 0.1 mg/kg, was selected, as the higher concentration with no toxicity, as shown below. (N=5).

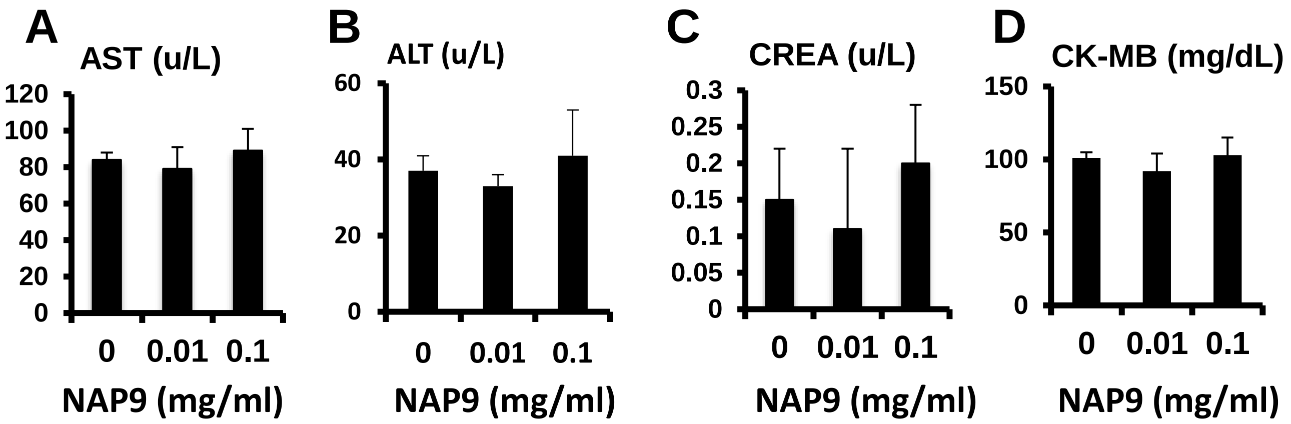

Figure S1. NAP9 cytotoxicity at different concentration (mg/ml). A) Aspartate transaminase (AST) **B)** Alanine transaminase (ALT), **C)** Creatinine (CREA) and **D)** creatine kinase MB (CK-MB) serum levels after NAP9 injection. N= 5 pigs.

Supplemental figures

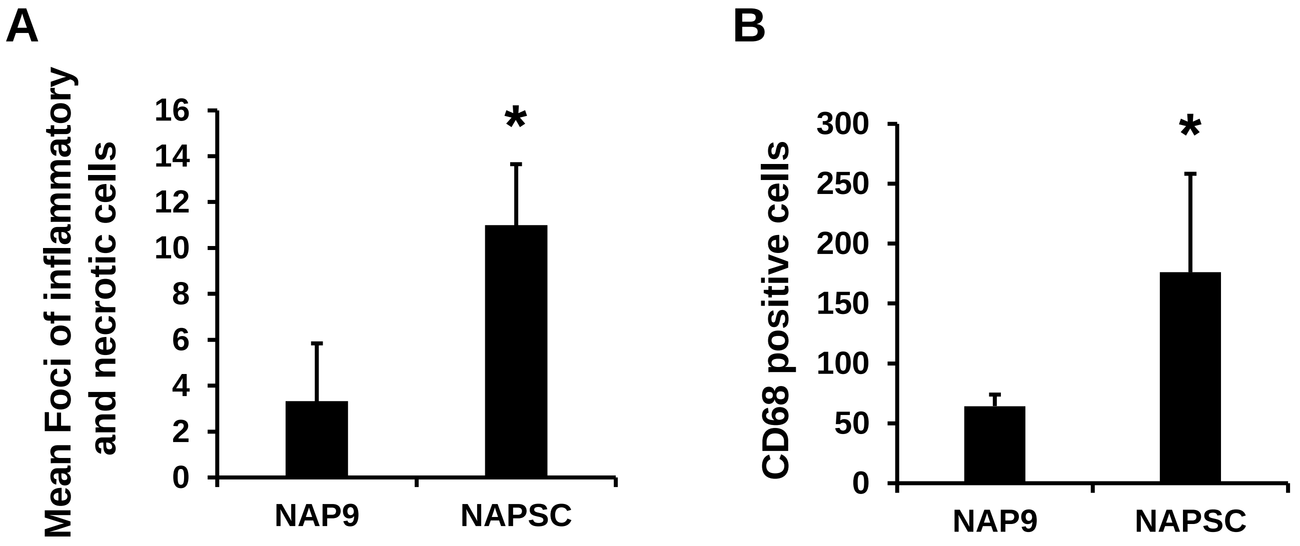

Figure S2. NAP9 reduces the number of necrotic and inflammatory cell foci detected after 7 days of IR. A) HE stained heart sections were used to count the total number of inflammatory and necrotic cell foci in the necrotic areas. Four necrotic heart sections/pig were used N= 5 pigs/group Mean± SD *$p < 0.05$. NAP9 vs. NAPSC. **B)** CD68 positive cells present in the necrotic areas. N= 5 pigs/group Mean± SD *$p < 0.03$. NAP9 vs. NAPSC.

Supplemental figures

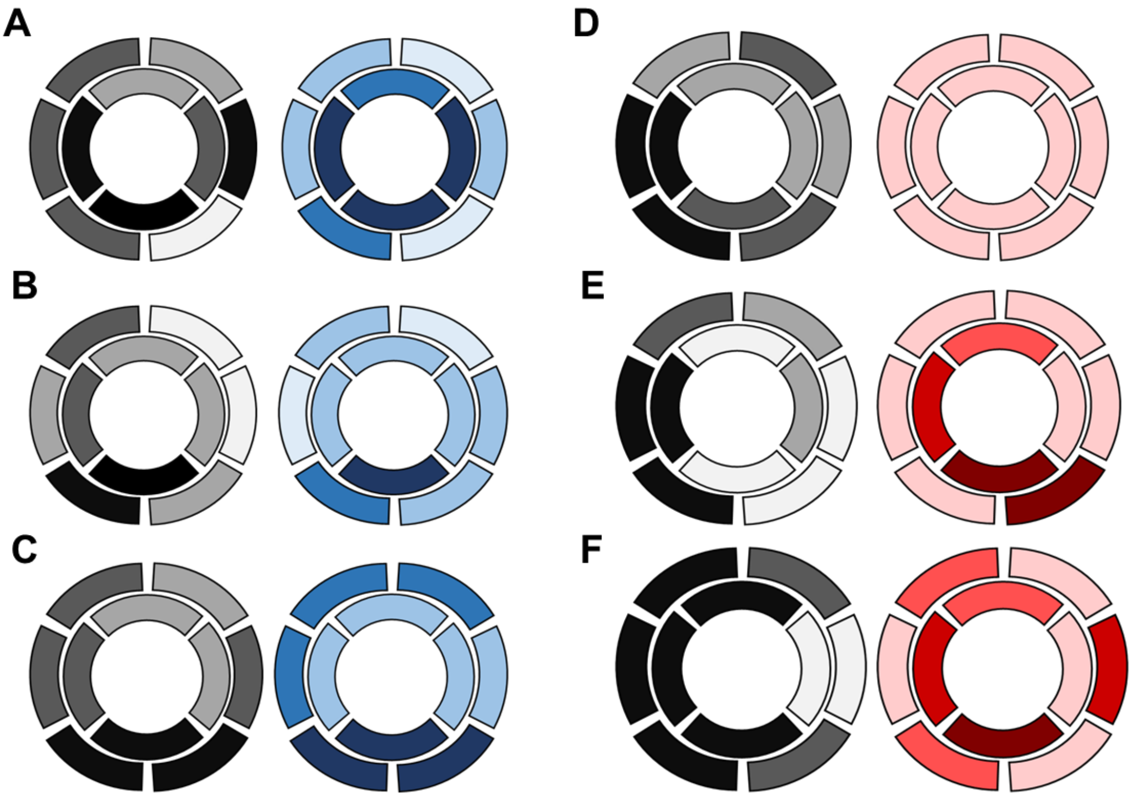

Native T1	ΔR1 NAP9
<1200	<4
1200<x<1225	4<x<5
1225<x<1250	5<x<6
>1250	>6

Native T1	ΔR1 NAPSC
<1200	<4
1200<x<1225	4<x<5
1225<x<1250	5<x<6
>1250	>6

Figure S3. Colored representation of Native T1 (grayscale) and ΔR1 (1/s) showing NAP9 concentration (blue scale) of myocardial sections. A) 1 day, **B)** 3 days or **C)** 7 days post-AMI. Colored representation of Native T1 (grayscale) and ΔR1 (1/s) showing NAPSC concentration (red scale) **D)** 1 day, **E)** 3 days or **F)** 7 days post-AMI.
